# Non-participation to a longitudinal and interventional survey on the psychological impact of the COVID-19 pandemic among healthcare workers (PSYCOVER) in France

**DOI:** 10.1192/j.eurpsy.2022.669

**Published:** 2022-09-01

**Authors:** C. Vuillermoz, L. Fossi, T. El Aarbaoui, A. Gosselin, N. Vignier, M. Melchior, S. Vandentorren, L. Bertuzzi

**Affiliations:** 1 Sorbonne Université, Inserm, Institut Pierre Louis D’épidémiologie Et De Santé Publique, Iplesp, Social Epidemiology Research Team, Paris, France; 2 Ined, Unité Mortalité Santé Epidémiologie, Aubervilliers, France; 3 Santé publique France, Direction Scientifique Et Internationale, Saint Maurice, France

**Keywords:** healthcare workers, Covid-19 pandemic, non-participation to a survey, longitudinal survey on mental health

## Abstract

**Introduction:**

We conducted a national longitudinal survey among healthcare workers in the context of the Covid-19 pandemic, (1) to assess mental health and (2) to describe the results of an intervention to improve capacity of resilience. Non-participation is rarely studied despite being an important methodological matter when performing studies on mental health.

**Objectives:**

The study aims to describe and identify the factors associated with non-participation of healthcare workers to the intervention part of a national longitudinal study on the psychological impact of the COVID-19 pandemic.

**Methods:**

Participants were recruited from April to October 2021 via an Internet link widely disseminated. Data collected include participant’ socio-demographic, occupational and working conditions, general health, professional burnout and mental health. The intervention proposed the use of tools for self-management of stress and resilience (PsySTART-Responder® and Anticipate.Plan.Deter™ program). A robust Poisson regression was used to identify factors associated with non-participation.

**Results:**

Among 724 participants, 41% participated to the intervention part. Factors associated to non-participation to the intervention were to work with few or no COVID-19 patients, and low scores in the anxiety scale. Social determinants, occupational characteristics or general health were not associated with non-participation.

**Conclusions:**

Our study provides a better understanding of the participation of healthcare workers that was not frequently studied. The results logically suggest lower participation among those with better mental health and not directly concerned with management of COVID-19 patients. Non-participation to the intervention was not associated with social factors, which is an argument in favour of using such a design/intervention in a socially heterogeneous population.

**Disclosure:**

No significant relationships.

